# Temporally optimized patterned stimulation (TOPS®) as a therapy to personalize deep brain stimulation treatment of Parkinson’s disease

**DOI:** 10.3389/fnhum.2022.929509

**Published:** 2022-08-24

**Authors:** Michael S. Okun, Patrick T. Hickey, Andre G. Machado, Alexis M. Kuncel, Warren M. Grill

**Affiliations:** ^1^Department of Neurology, Norman Fixel Institute for Neurological Diseases, University of Florida, Gainesville, FL, United States; ^2^Department of Neurology, Movement Disorders Center, Duke University Medical Center, Durham, NC, United States; ^3^Department of Neurology, Neurological Institute, Cleveland Clinic, Cleveland, OH, United States; ^4^Deep Brain Innovations, Cleveland, OH, United States; ^5^Department of Biomedical Engineering, Duke University, Durham, NC, United States

**Keywords:** Parkinson’s disease, deep brain stimulation, movement disorders, subthalamic nucleus, programming, stimulation parameters

## Abstract

Deep brain stimulation (DBS) is a well-established therapy for the motor symptoms of Parkinson’s disease (PD), but there remains an opportunity to improve symptom relief. The temporal pattern of stimulation is a new parameter to consider in DBS therapy, and we compared the effectiveness of Temporally Optimized Patterned Stimulation (TOPS) to standard DBS at reducing the motor symptoms of PD. Twenty-six subjects with DBS for PD received three different patterns of stimulation (two TOPS and standard) while on medication and using stimulation parameters optimized for standard DBS. Side effects and motor symptoms were assessed after 30 min of stimulation with each pattern. Subjects experienced similar types of side effects with TOPS and standard DBS, and TOPS were well-tolerated by a majority of the subjects. On average, the most effective TOPS was as effective as standard DBS at reducing the motor symptoms of PD. In some subjects a TOPS pattern was the most effective pattern. Finally, the TOPS pattern with low average frequency was found to be as effective or more effective in about half the subjects while substantially reducing estimated stimulation energy use. TOPS DBS may provide a new programing option to improve DBS therapy for PD by improving symptom reduction and/or increasing energy efficiency. Optimizing stimulation parameters specifically for TOPS DBS may demonstrate further clinical benefit of TOPS DBS in treating the motor symptoms of Parkinson’s disease.

## Introduction

Deep brain stimulation (DBS) of the subthalamic nucleus (STN) is a well-established therapy for the treatment of Parkinson’s disease (PD) (Benabid et al., 2009). The efficacy of DBS is highly dependent upon the programing of stimulation parameters, including the pulse amplitude, pulse duration, and pulse repetition frequency, and, the lack of understanding of the mechanisms of action has limited the optimization of this therapy.

High frequency (>100 Hz) stimulation has been overall more effective than low frequency DBS in treating tremor and most PD motor features (Rizzone et al., 2001; Moro et al., 2002; Kuncel et al., 2006). The basis for the frequency-dependent effects on symptoms was hypothesized to be related to the frequency-dependent regularization (or masking) of pathological neural activity (Birdno and Grill, 2008) and this is one of several hypothesized mechanisms of action of DBS (McIntyre et al., 2004; Herrington et al., 2016). In addition to the strong dependence on frequency, the effects of DBS on the motor symptoms of PD are also dependent upon the temporal pattern of stimulation (Grill, 2018). Random patterns of stimulation applied in PD patients, despite having the same high average frequency, were less effective at suppressing bradykinesia than regular patterns of stimulation (Dorval et al., 2010), and similar observations were made in patients with essential tremor (Birdno et al., 2008). The dependence of DBS efficacy on both the frequency and the temporal pattern of stimulation motivated the idea that the temporal pattern of DBS could be manipulated to increase the efficacy and efficiency of PD DBS. Indeed, acute, intraoperative assessment in subjects undergoing implanted pulse generator (IPG) replacement surgery (Swan et al., 2014) revealed that certain non-regular patterns of DBS treated bradykinesia more effectively than conventional high frequency DBS (Brocker et al., 2013) while a second pattern produced equivalent reductions in bradykinesia but greatly improved energy efficiency (Brocker et al., 2017).

A randomized prospective study was designed to determine the effects of Temporally Optimized Patterned Stimulation (TOPS®) on the motor symptoms of PD. This study was conducted across three centers for a longer duration than the intraoperative pilot studies. The study used custom firmware temporarily downloaded onto previously implanted IPGs to enable the delivery of novel stimulation patterns. A subject’s clinically optimized DBS parameter settings were used whenever possible. We evaluated two of the most promising patterns identified in the previous acute intraoperative study–designated TOPS1 and TOPS2–and compared the most effective TOPS to standard DBS and no stimulation.

## Materials and methods

This study was a prospective, randomized, cross-over, feasibility study. The effectiveness of two different non-regular temporal patterns of stimulation (TOPS1 and TOPS2) and standard DBS (sDBS) was compared with no stimulation (no stim).

### Study subjects

Individuals were recruited to participate in this study from the movement disorders centers at three sites including the Cleveland Clinic Foundation (Cleveland, OH), Duke University Medical Center (Durham, NC), and the University of Florida (Gainesville, FL). Eligible subjects had unilateral or bilateral STN DBS for Parkinson’s disease implanted at least 12 months prior and demonstrated DBS effectiveness.

The institutional review boards at the three sites approved the study protocol, and subjects enrolled after providing written informed consent and after verification of all eligibility requirements.

Twenty-six subjects (Table 1) with STN DBS (five unilateral, 21 bilateral) were enrolled. Motor data from 20 subjects, including 17 subjects with complete data sets (all outcome measures recorded for TOPS1, TOPS2, sDBS, and no stim) and three subjects with partial data sets (all outcome measures for sDBS, no stim, and either TOPS1 or TOPS2), were included in the statistical analysis of the effects of DBS on motor PD symptoms. For the three subjects with partial data sets, TOPS1 was not tested in one subject (A-02), and TOPS2 was not tested in two subjects (C-02, C-07) due to programing errors. Six of the 26 subjects were excluded from the motor symptom analysis due to missing motor response data for sDBS and/or both TOPS patterns. Missing motor response data were due to programing errors during testing (A-01, B-04, B-06, and C-04) or strong side effects experienced by the subjects (B-07, B-11).

**TABLE 1:** Subject characteristics.

Subject	Age (years)	Gender	Time since diagnosis (years)	Schwab and England score*	Hoehn and Yahr score*	Daily Levodopa equivalent dose (mg)
						
A-01	54.3	m	4.1	80	3	870
A-02	71.4	m	10.7	90	2	300
A-03	69.1	m	12.8	80	2	860
A-04	57.2	m	8.9	90	2	800
A-05	68.4	m	8.9	90	2	660
A-06	77.6	m	9.9	85	2	430
A-07	59.2	m	6.2	85	2	900
B-01	69.2	m	1.5	90	2	580
B-03	45.6	f	6.8	80	2.5	250
B-04	63.2	m	10.6	80	2.5	525
B-05	59	f	8.9	70	2.5	1000
B-06	63.3	m	5.8	90	2	0
B-07	59	m	9.1	90	2	0
B-09	72.6	m	11.5	90	3	400
B-10	63.1	m	6.2	100	3	50
B-11	67.4	f	16.6	90	3	1162.5
B-12^a^	57.6	m	6.1	100	2	0
C-01	54.9	m	n/a	90	2.5	1800
C-02	54.7	m	8.9	90	3	800
C-03	49	m	5.7	90	2	1280
C-04	41.6	m	4	90	2	500
C-05	63	f	18.8	80	2.5	570
C-06	67.1	m	6.2	80	3	800
C-07	64.5	m	9.1	90	2	800
C-08	62.8	m	10.9	100	2	560
C-09	61	m	22	60	2.5	550

*Assessments made while the subject was ON DBS and ON Parkinson’s medications.

^a^Subject took a Sinemet CR (25/100) as needed, on average once every 2–3-weeks.

n/a, data not available.

The mean age of the subjects was 61 ± 8.3 years (range 41–77 years) and, with DBS ON and on Parkinson’s medications, the mean Schwab and England Score was 86 ± 8.8 and the median modified Hoehn and Yahr Score was 2 (Table 1). Twenty-four of 26 subjects were taking Parkinson’s medications at the time of the study, and those subjects continued taking their regular medication doses throughout the pattern testing. Daily Levodopa Equivalent Doses are listed in Table 1. Subjects B-06 and B-07 were not taking Parkinson’s medications at the time of the study.

All subjects had a Medtronic Activa DBS system implanted with Medtonic electrodes (Model #3387 and Model #3389) at least 12 months prior to participation in this study with an average implant duration of 2.2 years at the time of the study (Table 2). Twenty-three of the 26 subjects demonstrated at least a 30% reduction in their UPDRS III motor score with DBS ON compared to no stim in the no medication state, and three subjects (A-03, A-05, and C-04) had reductions less than 30% (Supplementary Table 1). Six subjects (B-03, B-05, B-06, C-05, C-06, and C-08) had IPG replacements prior to study participation, ranging from 1.5 to 18 months prior. Clinical stimulation parameters are summarized in Table 2.

**TABLE 2:** Deep brain stimulation (DBS) parameters.

Subject	Time since implant* (years)	Right brain				Left brain			
		Contact configuration	Pulse width (μs)	Frequency (Hz)	Amplitude (V)	Contact configuration	Pulse width (μs)	Frequency (Hz)	Amplitude (V)
A-01	1.3	9-11+	60	180	3.9	1+2-3-	90	180	4.5
A-02^a^	3.3	2-C+	60	130	2.6	10-C+	60	130	2.6
		(2-3+)			(2.9)				
A-03	2.5	10-11+	90	130	4.5	2-3+	90	130	4.5
A-04^a,b^	1.3	1-2-3+	90	185	3.6	—	—	—	—
				(100)	(3.5)				
A-05	1.0	—	—	—	—	2-C+	60	130	3.4
A-06	1.6	10-11+	60	130	4.1	1 + 3-	90	130	3.7
A-07	1.5	—	—	—	—	2-C+	90	130	3.7
B-01^a^	2.7	10-C+	60	180	3.2	1-C+	60	180	3.2
						(2+3-)			
B-03^a^	2.6	9-10-C+	60	180	3	1-2-C+	60	180	3
						(1-2-3+)			
B-04^a^	1.2	10-11-C+	90	185	2.5	2-3-C+	90	185	2.5
		(9+10-11-)							
B-05^d^	7.0	9-10-11+	60	180	2.1	1-2-3+	60	180	2.2
B-06	3.1	8-9-10+	70	185	1.9	2-3-C+	70	185	3.8
B-07	1.6	8-9-10-11 +	90	185	4	0-1-2-3+	90	185	4
B-09^a^	1.1	10-C+	70	180	2.2	2-C+	70	180	2.3
					(2.8)	(2-1+)			(2.8)
B-10^a^	1.9	9-10-C+	80	185	2.4	1-2-C+	60	185	2.2
							(90)		
B-11^a^	1.2	9-C+	80	170	2.4	1-2-C+	90	170	2.7
						(1-2-3+)			
B-12^a^	1.2	8+9-10-11-	90	180	4.2	1-2-3-C+	90	180	3.4
		(9-10-11+)	(110)			(1-2-3+)			
C-01^a,b^	1.3	1-2-3+	150	180	2.7	9-10-11+	150	180	3
				(100)				(100)	
C-02^c^	2.5	n/a	n/a	n/a	n/a	1-3+	120	180	2
C-03	1.6	2-C+	120	185	2.7	—	—	—	—
C-04	1.6	—	—	—	—	1-C+	120	135	2.6
C-05^a^	6.1	10-C+	90	185	4	2-C+	90	185	4.1
		(9-10+)			(4.5)				
C-06^a^	3.1	2-C+	120	135	2	0-C+	90	200	3
						(0-1-C+)			
C-07	1.9	1-C+	120	100	3	1-2+	90	130	3
C-08	1.0	2-C+	120	180	1.7	1-3-C+	60	135	1.6
C-09^a^	2.7	1-C+	90	180	2	1-C+	120	180	2.8
				(135)	(2.1)				(2.5)

*If the subject had bilateral DBS with leads implanted on different dates, the time shown is from the second lead implant.

^a^Stimulation parameters changed from the clinical settings for pattern testing. The subject’s clinical settings are listed along with the settings used for testing, shown in parentheses.

^b^Stimulation frequency mistakenly programed to 100 Hz during standard pattern testing. The subject’s clinical stimulation frequency is listed.

^c^Receives bilateral stimulation clinically, but lead in right brain was connected to a Soletra IPG. Soletra IPG was turned OFF for study, and subject was tested unilaterally.

^d^Left lead was replaced 7 months prior to study participation, but stimulation parameters were steady at time of study.

n/a, data not available.

### Study design

Subjects were assigned to receive a sequence of test patterns of stimulation, and the order was randomized for each subject. The test patterns, including TOPS1, TOPS2, sDBS, and no stim, were tested during the same day in the medication “ON” state, and subjects were blinded to the pattern. The following three steps were repeated for each pattern. First, prior to turning each pattern on, stimulation was turned off for a 20-min washout period. Second, the Medtronic Activa IPG (bilaterally, if applicable) was programed to deliver the pattern and stimulation was initiated. Third, after 30 min of stimulation, while stimulation was still active, subjects rated the intensity of and described any side effects experienced during the 30 min of stimulation, a blinded evaluator administered part III of the Unified Parkinson’s Disease Rating Scale (UPDRS), and tremor (rest and postural) was recorded and quantified. These three steps were then repeated for each of the other three patterns.

The subject’s clinical stimulation parameters (i.e., contact configuration, pulse duration, and pulse amplitude; frequency when delivering standard DBS) that had previously been clinically optimized for sDBS were used during testing whenever possible. Reprograming was necessary in seven of the 26 subjects because the Research Programmer was unable to deliver stimulation with the same IPG (case) selected as the anode (+) bilaterally in subjects with a dual-channel implanted pulse generator. In these subjects, where the IPG (case) was set as the anode (C +) bilaterally, the side clinically programed with the lowest stimulation amplitude was reprogramed with an electrode contact (rather than the IPG) as the anode. In some instances, (3/7), additional reprograming of stimulation amplitude was done when deemed necessary by the clinician to optimize therapy after the change in contact configuration. Any changes made to stimulation parameters were made before testing began, were used during the testing of all patterns, and are listed in Table 2.

Subjects were evaluated while receiving a pattern while in the medication “ON” state. At the end of the 20-min washout from the previous stimulation, immediately before the next pattern was programed, the medication “ON” state was assessed by the clinician and by the subject completing the Wearing-Off-19 QUICK Questionnaire (WO19) (Martinez-Martin et al., 2008). “ON” state was defined as <2 symptoms on the WO19. If the results of either the WO19 or the clinician assessment suggested that the subject was in the “OFF” state, the clinician determined if the next dose of medication should be administered.

### Stimulation patterns

Temporally Optimized Patterned Stimulation (TOPS) are pulse trains with a repeating sequence of non-regular and non-random intervals between the stimulus pulses. TOPS1 was designed using model-based optimization with computational evolution and is a 9-pulse sequence with inter-pulse intervals of varying duration ranging from 2 to 52 ms. It has a lower average frequency (∼45 Hz) than the typical clinical frequency range of standard DBS (100–185 Hz), and therefore was hypothesized to be more efficient than standard DBS (Brocker et al., 2013). TOPS2 is a burst sequence composed of a long inter-burst interval (∼50 ms) followed by a burst with short IPIs (∼5 ms). TOPS2 has an average frequency (∼158 Hz) within the range of standard DBS, and prior intraoperative testing and computational analysis suggested TOPS2 to be more effective than standard DBS (Swan et al., 2014). The TOPS1 pattern may be found in Brocker et al. (2017) (“Genetic Algorithm Pattern”), and the TOPS2 pattern may be found in Brocker et al. (2013) (“Absence”).

To enable delivery of TOPS1, TOPS2, and sDBS, custom firmware was developed and temporarily downloaded to the IPG (Medtronic, Neuromodulation, Minneapolis, MN, United States). The firmware was compatible with Medtronic Activa PC, SC, and RC IPGs. Stimulation patterns were programmed by a trained clinician using a Microsoft Windows-based user interface running on a PC laptop connected to the IPG *via* a telemetry head (Medtronic, Neuromodulation). Due to the research programmer design, stimulation trains were delivered simultaneously to both hemispheres in subjects with bilateral electrodes and an Activa PC. Because of this, the research system only allowed one hemisphere to use the IPG as the anode (C +). All stimulation settings followed charge-balanced guidelines at or below standard clinical amplitudes and within all FDA safety guidelines (30 μC/cm^2^). At the conclusion of the testing session, temporary firmware was removed from all subject’s IPGs, and stimulation was restored to original clinically-optimal therapeutic settings.

### Outcome measures

Subjects rated the intensity of and described any side effects experienced during the 30 min of stimulation for each test pattern. Side effects were rated on a scale of 0–10, where a rating of 0 was no side effect and a rating of 10 was intolerable.

A blinded evaluator administered part III of the Unified Parkinson’s Disease Rating Scale (UPDRS III), and the assessment was video recorded. Scoring was completed by three independent raters, including the on-site blinded evaluator (Rater 1) and two remote raters (Raters 2 and 3) who provided scores after watching the video recordings of the assessments. Raters 2 and 3 imputed the rigidity scores recorded by Rater 1 since rigidity could not be assessed from the video. Motor symptoms were rated on a 0 to 4-point scale where 0 indicated “none” and 4 indicated “severe” symptom. The maximum total score for UPDRS III is 108, and the Minimally Clinically Important Difference (MCID) in UPDRS III is approximately 5.0 points (Hauser et al., 2014; Horvath et al., 2015). Motor subscores for tremor (Items 20–21), rigidity (Item 22), bradykinesia subscores (Items 23–26, and 31), and postural instability and gait disturbance (PIGD) subscores (Items 27–30) were calculated and analyzed.

Tremor was also quantified using a system of hand-mounted accelerometers and gyroscopes (Kinesia One System, Great Lakes Neurotechnology, Cleveland, OH, United States). The system quantified resting and postural tremor for the left and right upper limb, and the kinematic data were transformed into a score, ranging from 0 (no tremor) to 4 (severe tremor), using a validated algorithm (Giuffrida et al., 2009). Six subjects (A-04, A-05, A-07, C-02, C-03, and C-04) received unilateral stimulation during testing, and the tremor data from the upper limb opposite stimulation were included in the tremor analysis. In the remaining 14 subjects, data from the subjects’ more-affected side (assessed in the no stimulation condition) were included in the analysis. If the tremor with no stimulation was <0.05 (resting tremor *n* = 5, postural tremor *n* = 4), the subject was excluded from the analysis.

Estimated battery life was calculated according to the equations provided in Medtronic’s *System Eligibility, Battery Longevity* Manual (Medtronic, 2018) using the subject’s clinical settings at the time of the study. First, the clinical stimulation amplitude was rounded to the nearest volt, and Energy Usage was read from the “Activa PC and Activa SC IPG energy use for voltage mode” table. If the clinical stimulation frequency or pulse width was not listed in the table, the Energy Usage was interpolated for the clinical stimulation setting. For TOPS1, the Energy Usage for a frequency of 45 Hz was extrapolated from the data available in the table. Impedance was assumed to be 1,000 Ohms for all IPGs resulting in an impedance correction factor of one. The Adjusted Energy Use was calculated as Energy Use × the Impedance Correction Factor, and this number was doubled for subjects with an Activa PC. Finally, the longevity estimate was estimated from the Activa SC or PC IPG “longevity estimates (years) for energy use” figures.

### Data analysis and statistics

In some subjects, patterns were tested more than one time due to programing errors. In cases where test patterns were tested more than one time, the error was made in the testing sequence; the pattern testing was otherwise conducted according to protocol, and the evaluator remained blinded to the test pattern. In these cases, the repeated outcome measures were averaged. Motor response data were averaged for no stim in three subjects (A-02, B-09, and C-02) included in the motor analysis. Also, side effect intensity was averaged for no stim in five subjects (A-02, B-06, B-09, C-02, and C-04) and for TOPS1 in one subject (B-04) in the side effects results.

Side effects were recorded for each pattern tested (*n* = 26, No Stim; *n* = 23, standard DBS; *n* = 24, TOPS1; and *n* = 21, TOPS2). In some cases, side effect data were not recorded due to programing errors, due to missing data (A-05) or due to a pattern being skipped to avoid additional side effects (B-11).

Statistical analyses were conducted using JMP Pro 14 (SAS Institute, Inc., Cary, NC, United States). Motor outcome data for the most effective TOPS, sDBS, and no stim were analyzed using repeated measures analysis of variance (ANOVA) with UPDRS III scores or quantified tremor score as the repeated measure in each subject. *Post hoc* comparisons between stimulation patterns were made using Tukey’s Honestly Significant Difference test. UPDRS III scores were normally distributed, confirmed using the Shapiro-Wilk test. Battery life data did not meet the normality assumptions and a paired comparison was made using the non-parametric Wilcoxon Signed Rank test. Statistical significance was defined as α = 0.05.

## Results

The effects of different temporal patterns of STN DBS on the motor symptoms of Parkinson’s disease were quantified in a multi-center, randomized feasibility study conducted while patients were on their normal doses of Parkinson’s medications.

### Temporally optimized patterned stimulation reduced motor symptoms as effectively as clinically optimized standard deep brain stimulation

#### Unified Parkinson’s disease rating scale III

**FIGURE 1:**
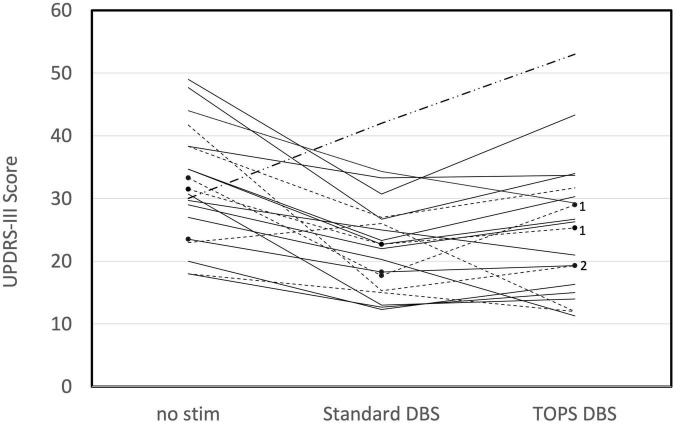
Motor symptoms, as measured using the UPDRS III in persons (*n* = 20) with PD and STN DBS while on their Parkinson’s medications are dependent on the stimulation pattern (One way ANOVA, *F* = 13.84, *p* < 0.0001). UPDRS III scores are shown for no stim, standard DBS (sDBS) and the most effective TOPS pattern (TOPS1 or TOPS 2). Patterns were tested with each patient’s stimulation parameters clinically optimized for sDBS. Dotted lines represent subjects in whom TOPS1 was the most effective TOPS pattern. Solid lines represent subjects in whom TOPS2 was the most effective TOPS. Note that TOPS1 and TOPS2 produced equal effects in one subject (B-05), for whom a dash-dot line is displayed. In three subjects, only one TOPS pattern was assessed, and these are marked with black dots and labeled with the pattern tested (TOPS1 = 1 or TOPS2 = 2).

Motor symptoms were quantified using UPDRS III scores during different patterns of DBS (Supplementary Table 2). Comparisons of UPDRS III scores between the most effective TOPS pattern (TOPS1 or TOPS2), sDBS, and no stim revealed that stimulation pattern had a significant effect on UPDRS III scores (One way ANOVA, *F* = 13.84, *p* < 0.0001) (Figure 1). UPDRS III scores were improved (reduced) compared to no stim with both sDBS (mean difference = 9.09, *p* < 0.0001) and the most effective TOPS (mean difference = 6.97, *p* = 0.0012). Also, UPDRS III scores in response to the most effective TOPS and sDBS were not significantly different (mean difference = −2.13, *p* = 0.47). Two subjects (A-05, B-05) had worsening of motor symptoms with sDBS compared to no stimulation, and this may be due to motor symptom fluctuation as a result of where they were in a medication cycle.

Comparison of the UPDRS III subscores for bradykinesia, tremor, and rigidity revealed stimulation pattern to have a significant effect on motor subscores (Table 3), with the exception of postural instability and gait disturbances. sDBS and the most effective TOPS produced equivalent reductions in UPDRS III subscores for tremor (mean difference = −0.8, p = 0.35), rigidity (mean difference = −0.5, *p* = 0.58), and bradykinesia (mean difference = 0.08, *p* = 0.99). Stimulation pattern did not have a significant effect on the PIGD subscores (*F* = 0.33, *p* = 0.72); neither sDBS nor TOPS DBS had a significant effect on the PGID motor subscores.

**TABLE 3:** Significance of stimulation patterns on UPDRS-III and motor subscores (*D* = mean difference).

	One-way ANOVA	*Post hoc* Tukey test results		
	Pattern significance	No stim vs. Standard DBS	No stim vs. TOPS DBS	Standard DBS vs. TOPS DBS
UPDRS-III Total	*F* = 13.84, *p* < 0.0001	*D* = 9.09, *p* < 0.0001	*D* = 6.97, *p* = 0.0012	*D* = −2.13, *p* = 0.47
Braykinesia	*F* = 7.72, *p* = 0.0015	*D* = 2.60, *p* = 0.005	*D* = 2.69, *p* = 0.004	*D* = 0.083, *p* = 0.99
Tremor	*F* = 13.24, *p* < 0.0001	*D* = 2.85, *p* < 0.001	*D* = 2.05, *p* = 0.0026	*D* = −0.8, *p* = 0.35
Rigidity	*F* = 16.64, *p* < 0.0001	*D* = 2.98, *p* < 0.0001	*D* = 2.43, *p* = 0.0002	*D* = −0.55, *p* = 0.58
PIGD	*F* = 0.33, *p* = 0.72	*D* = 0.27, *p* = 0.72	*D* = 0.20, *p* = 0.83	*D* = −0.067, *p* = 0.98

#### Tremor

**FIGURE 2:**
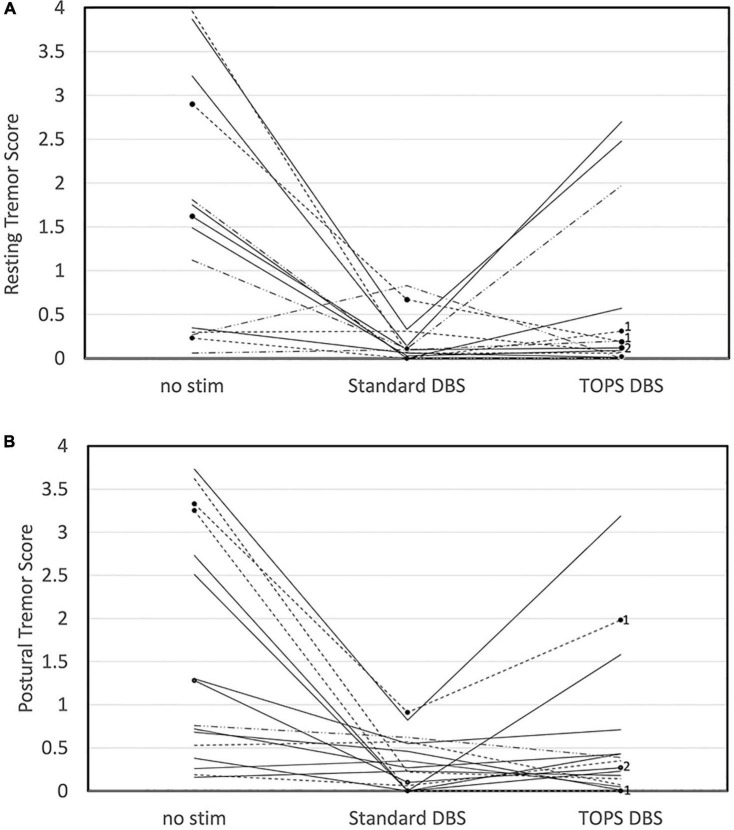
Tremor, quantified using the Kinesia One system, in persons with PD and STN DBS while on their Parkinson’s medications was dependent on stimulation pattern. Resting **(A)** and Postural **(B)** tremor scores are shown for no stim, sDBS and the most effective TOPS pattern (TOPS1 or TOPS 2). Patterns were tested with each patient’s stimulation parameters clinically optimized for sDBS. Scores varied across stimulation patterns for resting tremor (*F* = 9.64, *p* = 0.0007) and postural tremor (*F* = 11.64, *p* = 0.0002). Dotted lines represent subjects in whom TOPS1 was the most effective TOPS. Solid lines represent subjects in whom TOPS2 was the most effective TOPS. In three subjects, only one TOPS pattern was assessed, and these are marked with black dots and labeled with the pattern tested (TOPS1 = 1 or TOPS2 = 2).

Resting and postural tremors were quantified using a commercially available, objective, task-based motor assessment. Resting tremor was present (≥0.05) at baseline in 15/20 subjects and postural tremor in 16/20, and these subjects were included in the analysis. Comparisons of tremor scores between the most effective TOPS pattern (TOPS1 or TOPS2), sDBS, and no stim revealed that stimulation pattern had a significant effect on resting tremor (One-way ANOVA, *F* = 9.64, *p* = 0.0007) (Figure 2A) and postural tremor (*F* = 11.64, *p* = 0.0002) (Figure 2B).

Resting tremor scores were improved (reduced) compared to no stim with both sDBS (mean difference = −1.34, *p* = 0.0006) and the most effective TOPS (mean difference = −0.95, *p* = 0.014), and resting tremor scores in response to the most effective TOPS and sDBS were not significantly different (mean difference = −0.40, *p* = 0.43). Similarly, postural tremor scores were improved compared to no stim with both sDBS (mean difference = −1.27, *p* = 0.0002) and the most effective TOPS (mean difference = −0.96, *p* = 0.004). Postural tremor scores in response to the most effective TOPS and sDBS were not significantly different (mean difference = −0.30, *p* = 0.52).

### TOPS deep brain stimulation was more effective than standard deep brain stimulation in a subset of subjects

**FIGURE 3:**
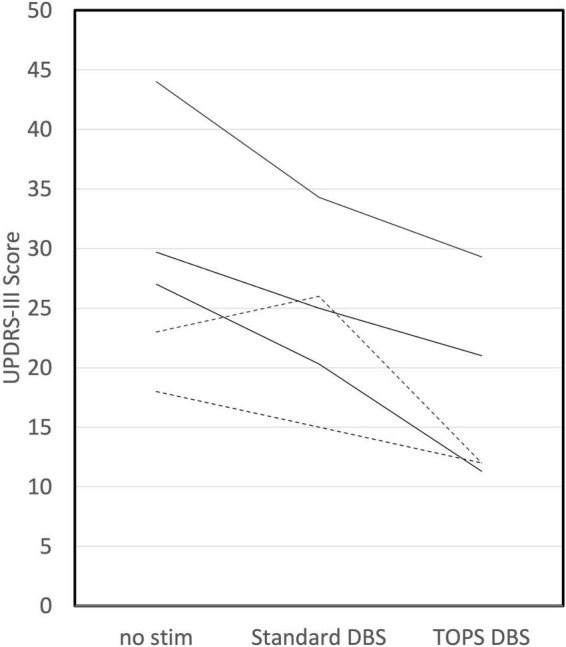
TOPS DBS reduced UPDRS III (improved symptoms) more than standard DBS in 25% (5/20) of subjects. Dotted lines represent subjects in which TOPS1 was the most effective TOPS. Solid lines represent subjects in which TOPS2 was the most effective TOPS.

Temporally optimized patterned stimulation DBS reduced the UPDRS III more than sDBS in 25% (5/20) of subjects (Figure 3). Further, in three of those five subjects, TOPS DBS reduced UPDRS III by at least five points more than sDBS. Conversely, sDBS reduced the UPDRS III more than TOPS DBS in 75% (15/20) of subjects. Further, in five of those 15 subjects, sDBS reduced UPRS III by at least five more points than TOPS.

Similarly, TOPS DBS was more effective than sDBS at reducing tremor in a subset of subjects. TOPS DBS improved the tremor score more than sDBS in 33% (5/15) of subjects for resting tremor and 38% (6/16) for postural tremor. Meanwhile, sDBS improved the tremor score more than TOPS DBS in 47% (7/15) subjects for resting tremor and in 46% (9/16) for postural tremor. When comparing no stim and the most effective TOPS, resting tremor increased with the TOPS DBS in three subjects. In two of these subjects (A05, C02), the resting tremor was rated less than 0.5 (out of 4) for both no stim and the most effective TOPS. In the third subject (C08) tremor increased from 1.1 with no stim to 2 with most effective TOPS.

### TOPS1 deep brain stimulation was as effective as standard deep brain stimulation in a subset of subjects and used substantially less energy

**FIGURE 4:**
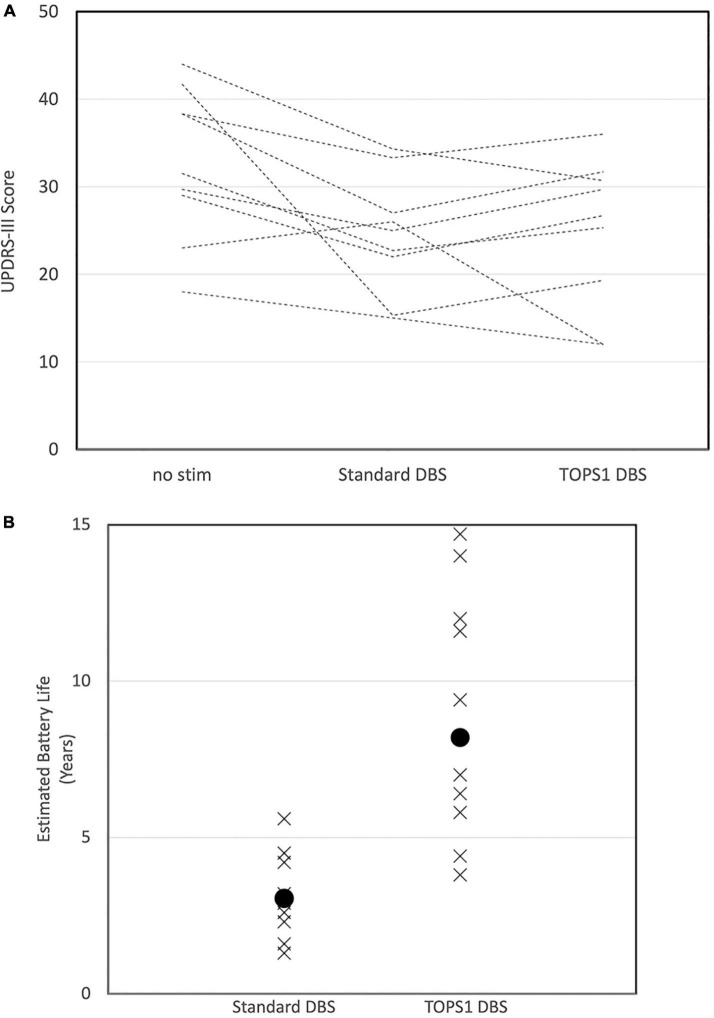
Motor scores (UPDRS III) in subjects (*n* = 9/19) for whom TOPS1 (average frequency = 45 Hz) was as effective or more effective than sDBS **(A)**. Subjects all have PD and STN DBS and were tested while on Parkinson’s medications. Estimated implanted pulse generator battery life for sDBS and TOPS1 DBS **(B)** in nine subjects in which TOPS1 was as effective or more effective than sDBS. Median estimated battery life was significantly increased with TOPS1 DBS (*S* = 27.5, *p* = 0.002).

TOPS1 was tested in 19 of the 20 subjects included in the motor symptom analysis, and TOPS1 was as effective (<5 point difference) or more effective at reducing the UPDRS motor score than sDBS in 47% (9/19) of subjects (Figure 4A). sDBS clinically improved (>5 point reduction) motor symptoms in 79% (15/19) of subjects and TOPS1 clinically improved motor symptoms in 42% (8/19) of the subjects. TOPS1 clinically improved motor symptoms in two subjects that sDBS did not; conversely, sDBS clinically improved motor symptoms in nine subjects that TOPS1 did not. When considering tremor, sDBS and TOPS1 reduced resting tremor in 71% (10/14) of subjects. sDBS and TOPS1 reduced postural tremor in 80% (12/15) and 67% (10/15) of subjects, respectively. TOPS1 was as effective as or more effective than sDBS at reducing tremor in 43% (6/14) of subjects for resting tremor and in 20% (3/15) of subjects for postural tremor. sDBS was as effective or more effective than TOPS1 at reducing rest tremor in 64% (9/14) of subjects and in 87% (13/15) of subjects for postural tremor.

In the nine subjects for which TOPS1 (average frequency = 45 Hz) was as effective or more effective than sDBS, at the battery life of each subject’s IPG was estimated (Medtronic, 2018) for sDBS and TOPS1 using the subject’s clinically optimized parameters for sDBS (Figure 4B). Median estimated IPG lifetime was significantly longer with TOPS1 (median = 8.2 years) than for sDBS (median = 3.0 years) (Wilcoxon Signed Rank Test, S = 27.5, *p* = 0.002). The median estimated IPG lifetime for TOPS2 (average frequency = 158 Hz) was 3.2 years.

### Side effects elicited with temporally optimized patterned stimulation and standard deep brain stimulation were comparable

**FIGURE 5:**
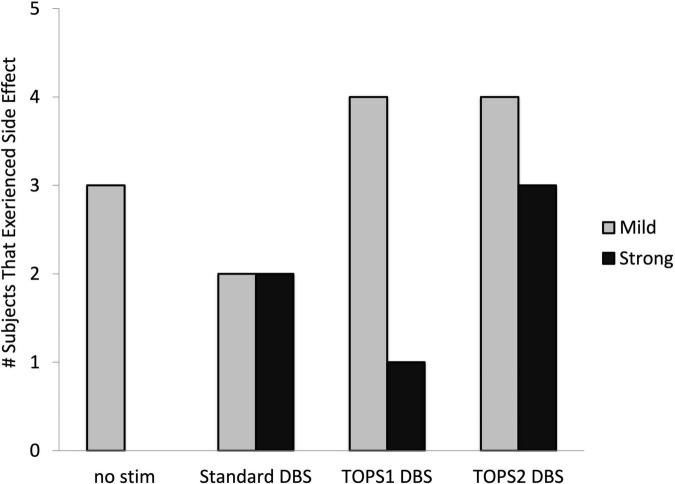
Number of subjects who experienced side effects with each stimulation pattern. Side effects with a mild intensity were rated less than an eight. Side effects with strong, sustained intensity and considered intolerable were rated 8, 9, or 10. Side effects were assessed in *n* = 26 (no stim), *n* = 23 (sDBS), *n* = 24 (TOPS1), and *n* = 21 (TOPS2) out of 26 subjects.

Eleven of the 26 subjects experienced a side effect during stimulation with one or more stimulation patterns. The types of side effects were similar across both TOPS patterns and sDBS and included paresthesias, difficulty speaking, involuntary muscle contractions, vision changes, and sensations of coldness, sweating, and anxiety. Side effects were reported 19 times across 11 subjects (Figure 5). There were 13 occurrences where the test patterns elicited mild and transient (rated < 8) side effects and six where strong and sustained (rated ≥ 8) side effects were elicited and required stimulation be turned off before the end of the 30-min test duration and precluded motor testing. TOPS1 (*n* = 1), TOPS2 (*n* = 3) and/or standard DBS (*n* = 2) elicited strong and sustained side effects in four subjects (Table 4).

**TABLE 4:** Strong, sustained side effects were experienced by four subjects during at least one test pattern resulting in stimulation being turned OFF before the 30-min test period.

Subject	No stim	Standard	TOPS1	TOPS2
B-06	0	n/a	0	9
B-07	1	10	1	10
B-11	0	10	8	n/a
C-07	0	0	5	9

Strong side effects were defined as those rated 8–10 and are highlighted in gray. n/a: data point not available; pattern was skipped to avoid side effect.

## Discussion

This multicenter study demonstrated the safety and feasibility of TOPS DBS as a novel approach to neuromodulation therapy. The data revealed a potential for TOPS DBS to improve the motor symptoms of PD more effectively and/or more efficiently than sDBS in a subset of patients. Patients with different Parkinson’s disease subtypes may respond differently to TOPS DBS, but this study focused on feasibility and was not designed to evaluate effects on specific symptoms. Consistent with intra-operative testing of TOPS (Brocker et al., 2013, 2017), these novel patterns were well-tolerated by most subjects, and they were effective in alleviating the motor symptoms of PD. There were several key differences between this study and previous testing of TOPS DBS (Brocker et al., 2013, 2017): in the previous study testing was intraoperative, bradykinesia was estimated using a finger-tapping task on a computer mouse, the stimulation duration prior to completing a motor assessment was about 2–4 min of stimulation, and subjects were asked to withhold their Parkinson’s medications. For the current study, TOPS settings were implemented in subjects with previously-implanted IPGs using a non-invasive firmware download. Firmware updates such as TOPS have the potential to provide a more personalized treatment for DBS patients, and device updates may enable such stimulation capabilities in the future.

Overall, TOPS reduced the motor symptoms of Parkinson’s disease to the same degree as sDBS. The mean UPDRS III score for the most effective TOPS setting (either TOPS 1 or 2) was not significantly different than that with sDBS, and both TOPS and sDBS alleviated motor symptoms from the no stim condition. Similarly, the reduction in the bradykinesia, tremor, and rigidity subscores was similar between sDBS and the most effective TOPS. Further, the results of the quantitative measurement also demonstrated that sDBS and TOPS both reduced postural and resting tremor.

Temporally optimized patterned stimulation DBS reduced the UPDRS III more than sDBS in 25% (5/20) of subjects. TOPS1 DBS reduced UPDRS III maximally in two subjects and TOPS2 reduced UPDRS III maximally in three subjects. Importantly, TOPS2 reduced Parkinson’s symptoms in one subject in which sDBS failed. Two subjects (A-05, B-05) had worsening of motor symptoms with sDBS compared to no stim, and this may be due to motor symptom fluctuation because of where they were in a medication cycle. Subjects A-05 and B-05 were taking frequent doses of medication (5×/day and 8×/day, respectively) and therefore likely had short ON/OFF cycles. TOPS DBS was also effective at reducing postural and resting tremor. Of note, the stimulation parameters used during testing of different stimulation patterns were optimized for sDBS. It is possible that optimization of contact configuration, pulse width, and pulse amplitude for TOPS would further increase the number of responders and the degree of symptom suppression relative to sDBS.

TOPS1 was as effective or more effective than sDBS at improving motor symptoms in almost half of subjects (9/19) and had the benefit of potentially doubling median IPG lifetime due to its lower average frequency. Current primary cell powered IPGs require surgical replacement every 3–5 years for standard stimulation settings (Medtronic, 2018), and increasing IPG lifetime will reduce the risks associated with device replacement including acute loss of symptomatic relief and infection as well as reduce the overall cost of DBS therapy by reducing the number of required replacements during a patient’s lifetime. In IPGs powered by rechargeable batteries, TOPS1 will increase the time between required recharging. The energy savings from TOPS1 could be most impactful for patients receiving DBS for applications requiring higher voltages (currents). Alternatively, the energy savings from TOPS1 could be directed to reduce the volume of IPGs and to maintain current device lifetime and recharge intervals.

The TOPS patterns were well-tolerated by a majority of the subjects and the types of side effects experienced were similar between sDBS and the TOPS patterns. Mild, transient side effects were experienced across all test patterns, including with the no stim condition. Four subjects rated the side effect intensity as strong, lasting, and intolerable (8, 9, or 10), but the intolerable side effects were not unique to TOPS. Two subjects (B-07, B-11) who rated TOPS patterns as intolerable also rated the side effects of sDBS as intolerable. Both subjects were receiving bilateral stimulation from a single IPG. A possible explanation for strong side effects seen during sDBS is that for subjects with bilateral stimulation, pulses were delivered simultaneously to both hemispheres, instead of alternating pulses between hemispheres (interleaving) as standard for clinical therapy. Simultaneous bilateral stimulation may have also played a role in the intolerable side effects elicited by TOPS in one subject (B-06) in whom sDBS was not tested. The fourth subject who experienced intolerable side effects (C-07) was programed clinically to receive 100 Hz stimulation. The stronger side effects elicited with TOPS2 may have been caused by the increase in average frequency, and this may have been alleviated by a reduction in stimulation amplitude. Because this study was conducted as a clinical trial, stimulus parameter settings were not permitted to be adjusted during testing. Such adjustments usually can be used to increase tolerability.

This was a small feasibility study with a limited number of subjects meant to demonstrate tolerability and effectiveness of TOPS after a longer period (∼30 min) of stimulation and to inform the design and powering of a subsequent study. There were several important limitations to this study. Most important was that the patterns were not tested in a chronic state after several days on a particular setting. The effects of the different patterns on motor symptoms may change over a longer period of stimulation, and further adjustments to stimulation parameters or medications may be required. Assessments made at the 30-min mark are like those made during clinical programing, where the parameters are selected and determined to be appropriate to take home after a relatively short epoch of stimulation.

Also, an important purposeful design of this study was that subjects were tested while ON their Parkinson’s medications, and this facilitated comparison of patterns in the subjects’ best clinical state. This also allowed the testing session to be more tolerable for subjects and allowed us to determine whether switching to TOPS may be feasible for the many patients with pre-existing DBS devices. However, it is an important limitation that the timing of medication dosing was not consistent across subjects and that motor fluctuations due to medication status may have increased the variance in the assessments of the effects of stimulation patterns. The medication “ON” state was assessed by a clinician and also by the subject who completed the Wearing-Off-19 QUICK Questionnaire. The WO19 was used to screen for potential wearing off of medications, and the clinicians rendered a bedside decision if administering a next dose of medication was appropriate to maintain the patient in an ON medication condition.

Another limitation of the study was the heterogeneous nature of the subjects’ electrode configurations (bilateral vs. unilateral, monopolar vs. bipolar). Finally, and perhaps most importantly, the programmers in the study were not permitted to optimize the stimulation settings for TOPS. Based on our experience with DBS therapy, it would be likely that slight individual modifications could lead to even more benefits. It was assumed that subjects’ standard DBS settings were reasonably optimized prior to the study.

This was the first test of TOPS in the clinical setting, and the results demonstrated that novel patterns of stimulation could provide a useful alternative to standard DBS. TOPS delivered a more efficient and for some patients a more effective option for DBS treatment. These patterns provide an entirely new parameter space for optimization of DBS for Parkinson’s treatment and possibly could be used in other applications of DBS (Grill, 2018). Other recent studies have also demonstrated the feasibility of patterned DBS to improve DBS therapy by increasing the therapeutic window (Horn et al., 2020) or by improving axial symptoms in a subset of subjects (Sáenz-Farret et al., 2021). We posit that TOPS will be an important step for the personalization of the DBS experience which should be aimed at improving the outcome for individual patients with unique symptom profiles.

## Data availability statement

The raw data supporting the conclusions of this article will be made available by the authors, without undue reservation.

## Ethics statement

The studies involving human participants were reviewed and approved by Duke Health Institutional Review Board, Cleveland Clinic Institutional Review Board, and the WCG Institutional Review Board. The patients/participants provided their written informed consent to participate in this study.

## Author contributions

MO: research project conception, study design and execution, and review of statistical analysis and the manuscript. PH and AM: study execution and review of the manuscript. AK: study design, project organization and execution, execution and review of statistical analysis, and writing first draft of manuscript. WG: research project conception, study design, and review of statistical analysis and manuscript. All authors contributed to the article and approved the submitted version.

## Abbreviations

DBS: deep brain stimulation
STN: subthalamic nucleus
PD: Parkinson’s disease
IPG: implanted pulse generator
TOPS: temporally optimized patterned stimulation
WO19: wearing-off-19 QUICK questionnaire
UPDRS: unified Parkinson’s disease rating scale
MCID: minimally clinically important difference
PIGD: postural instability and gait disturbance
ANOVA: analysis of variance
sDBS: standard DBS.
