# Supplementary material for: Temporally optimized patterned stimulation (TOPS®) as a therapy to personalize deep brain stimulation treatment of Parkinson’s disease
Source: Front Hum Neurosci. 2022 Aug 24;16:929509. doi: 10.3389/fnhum.2022.929509 (PMC9454097; doi:10.3389/fnhum.2022.929509)
Supplement: Supplementary file 1 [file Table_1.docx]

**Supplementary Table 1**: Pre-Study Reduction in UPDRS III in the off-medication state

Note: Site A used the pre-operative UPDRS III score in the off-medication state and the ON DBS, off medication score to calculate the % Reduction. Sites B and C used the OFF DBS UPDRS III score.
