# Supplementary material for: Temporally optimized patterned stimulation (TOPS®) as a therapy to personalize deep brain stimulation treatment of Parkinson’s disease
Source: Front Hum Neurosci. 2022 Aug 24;16:929509. doi: 10.3389/fnhum.2022.929509 (PMC9454097; doi:10.3389/fnhum.2022.929509)
Supplement: Supplementary file 2 [file Table_2.docx]

**Supplementary Table 2**: UPDRS III scores in persons (n=20) with PD and STN DBS, in the medication “ON” state and in response to no stim, standard DBS (sDBS), TOPS1 DBS, and TOPS2 DBS. Patterns were tested with each patient’s stimulation parameters clinically optimized for sDBS. UPDRS III scores shown are the average of three scores, including the on-site blinded evaluator (Rater 1) and two remote independent raters (Raters 2 and 3) who provided scores after watching the video recordings of the assessments. n/a: score not available
